# How Refined Surgical Technical Solutions Can Make Bentall Operation a Low-Risk Procedure: 20-Year Personal Experience at the “Root” of the Aortic Diseases—It Is Time to Change Surgical Guidelines

**DOI:** 10.3390/jcm12237330

**Published:** 2023-11-26

**Authors:** Giovanni Ruvolo, Claudia Altieri, Carlo Bassano, Dario Buioni, Paolo Nardi, Calogera Pisano

**Affiliations:** 1 Department of Cardiac Surgery, Tor Vergata University of Rome, 00133 Rome, Italy; giovanni.ruvolo@uniroma2.it (G.R.); carlo.bassano@uniroma2.it (C.B.); docyuk@libero.it (D.B.); pa.nardi4@libero.it (P.N.); 2 Cardiology Unit of the Cardiac Surgery Division, Tor Vergata University of Rome, 00133 Rome, Italy; claudia.altieri@ptvonline.it

**Keywords:** ascending aorta aneurysm, Bentall–De Bono operation, button technique, surgical guidelines

## Abstract

(1) Objective: Twenty years’ experience of Bentall–De Bono operations by one surgeon. (2) Methods: From January 2003 to September 2023, four-hundred-and-two patients aged 65.9 ± 15 years underwent a Bentall operation. The EuroScore-2 was 5.0% ± 3.8%. Associated procedures were performed on 113 patients (28.1%). Results: Operative mortality was 1.2% (*n* = 5), in particular 0.69% (*n* = 2/289) for isolated Bentall operation, 2.65% (*n* = 3/113) for combined procedures (*p* < 0.05). Postoperative acute heart failure occurred in 38 patients (9.45%). Preoperative pulmonary hypertension (44 ± 14 vs. 33 ± 7 mmHg), cardiopulmonary bypass time (169 ± 61 min. vs. 124 ± 42 min.) and aortic cross-clamp time (133 ± 45 min. vs. 107 ± 34 min.) have been recognized as independent predictors of mortality and cardiac complications (*p* < 0.05). Conclusions: In our experience, the Bentall operation was associated with low operative mortality and low rate of complications. For this reason, in agreement with the patients, we have modified surgical indication for ascending aortic aneurysms and now we think that it is time to change surgical guidelines.

## 1. Introduction

Since its introduction, the Bentall–De Bono operation became the best treatment for ascending aorta pathology and is now the most-used procedure [1]. It is adopted by many cardiac surgeons with good clinical results [2,3,4,5,6,7,8,9,10]. In our opinion, specific expedients in this surgical technique can greatly improve the results. In this study, we described the surgical technical aspects in the Bentall–De Bono operation during personal (G.R.) twenty-year surgical experience. We also reviewed early surgical outcomes of Bentall operation performed according with our indications.

## 2. Materials and Methods

### 2.1. Study Population

We enrolled 402 patients (320 males, 82 females; mean age 65.9 ± 15.0 years) who suffered from ascending aorta aneurysm (mean diameter 50.0 ± 7.3 mm) or aortic root aneurysm (mean diameter 44.5 ± 6.6 mm) and underwent a Bentall operation. All the procedures were performed by a single operator (G.R.) at Palermo University Hospital first and at Tor Vergata University Hospital later, from January 2003 to September 2023. The EuroScore-2 was 5.0% ± 3.8%. In 238 patients (59%), the ascending aorta aneurysm was associated with a moderate or severe degree of aortic valve regurgitation or combined stenosis and regurgitation. Tricuspid aortic valve was detected in 221 patients (55%), bicuspid valve in 161 patients (40%). Combined procedures (coronary artery bypass grafting, mitral valve repair, or replacement with or without tricuspid valve repair, closure of atrial septal defects, aortic arch replacement) were performed in 113 patients (28.1%). Patients operated on for acute aortic syndromes and acute endocarditis were not included in the present study. The study was a retrospective one. It was performed according to the Declaration of Helsinki and approved by the Independent Ethic Committee of the Tor Vergata University Polyclinic (Protocol Title: 01-Aorta-2018; Protocol Number: 179/18). All patients gave their informed surgical consent. 

### 2.2. Data Analysis

We analyzed the following preoperative variables: age, gender, high blood pressure, hypercolesterolemia, smoking, diabetes, obesity, myocardial infarction, atrial fibrillation, echocardiographic parameters, i.e., left ventricular hypertrophy, thickness of the septum and the posterior wall, end-diastolic and end-systolic diameters, pulmonary arterial pressure, left ventricular ejection fraction, left atrial dimensions, type of aortic valve pathology. Intraoperative data were also collected, i.e., cross clamp time, cardio-pulmonary bypass time, and associated procedures. In-hospital mortality, mortality within 30 days after discharge, postoperative acute heart failure combined with or without acute renal dysfunction, and need for early surgical re-exploration for bleeding and for pacemaker implantation were investigated.

### 2.3. Surgical Indications

**Figure 1:**
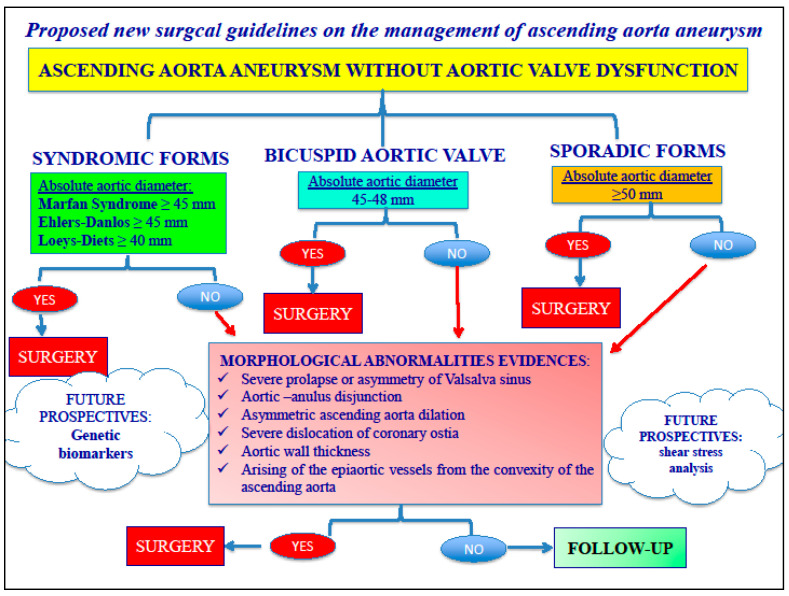
Proposed management of ascending aorta aneurysm.

According with our experience [11,12,13,14,15,16], surgical indication was based not only on a smaller diameter (≥5.0 cm for sporadic thoracic aortic aneurysms (TAA) and ≥4.5 for syndromic TAA with or without aortic valve dysfunction) than that advocated by the EACTS/ESC guidelines [17], but also on etiological parameters and on morphological features, like coronary ostia dislocation and prolapse, quality of the aortic wall, aortic–annulus disjunction, i.e., asymmetric dilation of Valsalva sinus/sinuses [15] (Figure 1). 

Obviously, preoperatively, all patients gave their surgical consent after being informed about the differences between the international guidelines and our different personal surgical indications. 

### 2.4. Surgical Technique

The aforementioned technical details represent only suggestions dictated by one’s personal experience of 402 modified Bentall–De Bono operations, performed electively or/and urgently using composite graft valved conduits with mechanical prostheses (56%) or using a tubular cylinder graft (44%) assembled with a last-generation biological aortic valve prosthesis. All patients underwent median sternotomy. 

**Figure 2:**
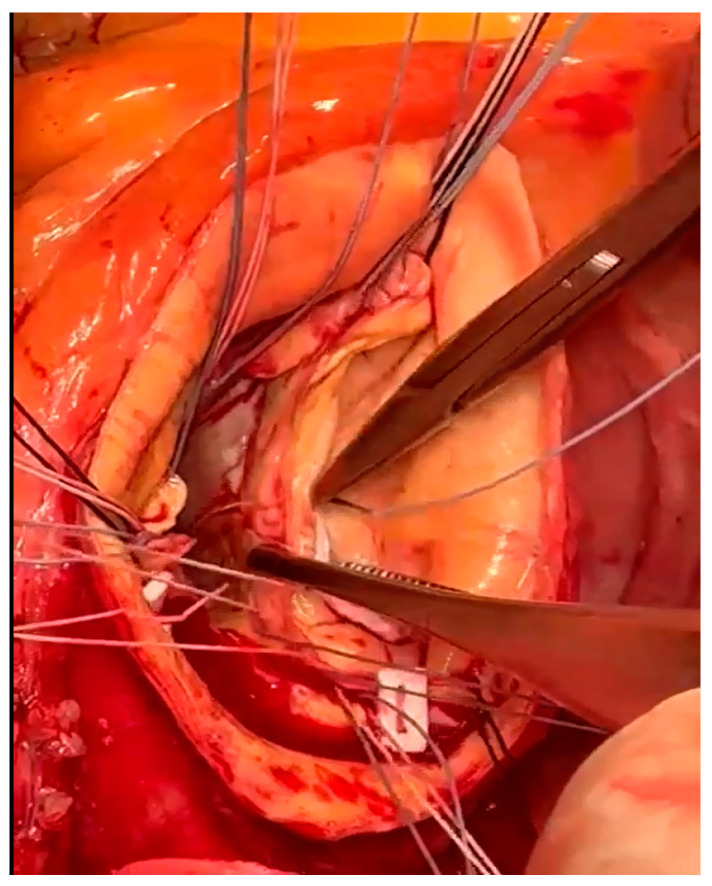
Valvular stitches at the level of the non-coronary sinus.

**Figure 3:**
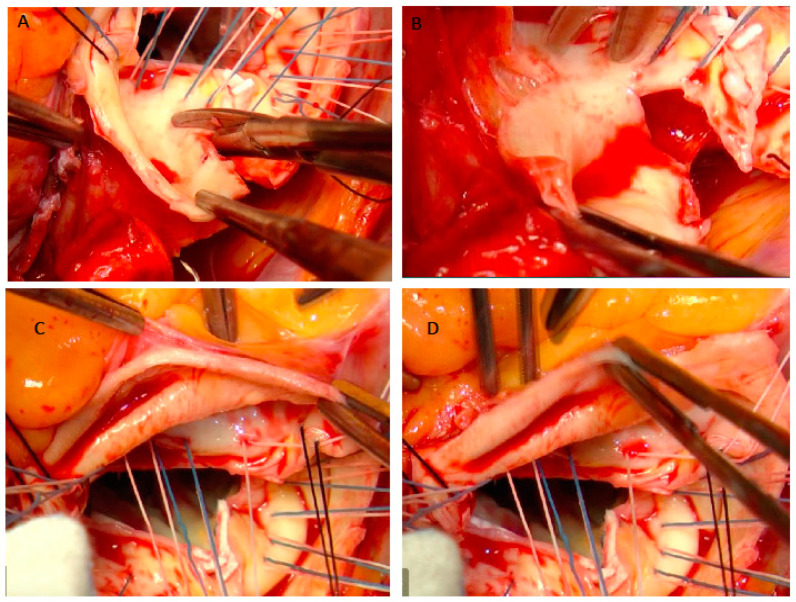
Preparation and excision of the left coronary ostium. Preparation of the inferior margin of the left coronary ostium (**A**,**B**). Total excision of the left coronary ostium (**C**,**D**).

Systemic heparinization is followed by cannulation of either the proximal aortic arch or the femoral artery. Aortic arch cannulation is performed using a specific arterial cannula through a double-pledgeted purse-string suture. In case of aortic arch pathology, a femoral artery cannulation is performed. Right atrium is routinely used for venous cannulation. A venting cannula is placed into the right upper pulmonary vein to decompress the left heart. We usually place the ventricular vent before the aortic cross-clamping, in order to decompress the left ventricle during aortotomy. The ascending aorta is clamped just below the brachiocephalic artery. After ascending aorta cross-clamping, the aorta is transected approximately 1.5 cm above the fat covering the anterior aortic root in order to avoid the right coronary ostium transection, in particular in cases of anteriorly coronary ostium dislocation. St. Thomas crystalloid cardioplegic solution is administered intermittently and antegrade selectively through the coronary ostia. After cardioplegia, the ascending aorta and the aortic valve leaflets are resected in a fashion that left at least 0.8 cm of native aortic tissue around the aortic clamp and 0.5 cm around the coronary ostia, respectively. Being careful not to damage the right pulmonary artery branch, the posterior and medial wall of the ascending aorta is separated from the underlying pulmonary artery using the electric scalpel. Subsequently, three suspension stitches are passed to the commissures in order to improve the surgical view and facilitate the passage of valvular stitches. The composite graft is sutured to the aortic annulus with pledgeted tapering 2-0 polyester sutures. The valvular stitches are passed in a subannular position or in a supra-annular position according with the aortic annulus size. We prefer the supra-annular position of the pledgets because we assume that blood flow through the left ventricular outflow is better. In both cases we start to pass the valvular stitches counterclockwise, starting from the commissure between the non-coronary sinus and the right-coronary sinus. When we use the sub-annular technique, we paid attention: (a) to pass the valvular stitch very close to the aortic annulus to avoid injuring the bundle of His; (b) at the level of the noncoronary sinus, not to pass the stitches close to the anterior mitral leaflet to avoid its tearing and/or its damage (Figure 2); (c) at the level of the left-coronary sinus, not to pass the stitches too close to the edge of the aortic annulus and, at the same time, to leave an adequate amount of aortic wall to perform the suture of the ostium on the vascular conduit. At this point, the optimal sizes of the prosthetic valve and graft (bio-Bentall) or of the valved conduit (mechanical Bentall) are determined. In our opinion, in order to choose the correct size of the valve prosthesis, the sizer should almost slide along the wall of the aortic annulus without any obstacle. We use this technical expedient because, after cardiac arrest, it may be induced to overestimate the size of the prosthetic valve to be implanted. Usually, we assemble ourselves the conduit for the bio-Bentall operation using a vascular graft 3 mm larger than the biological valve prosthesis. We prefer a straight and rigid vascular conduit (Intervascular, Datascope Corp., Wayne, NJ, USA) because, in our opinion, it is easier to adapt it to the coronary ostia, both during the preparation of the anastomosis and during the dilation of the conduit after aortic declamping. Using a hand-held cautery device, the coronary ostia are excised together with a ‘collar’-like remnant of the aortic wall, extending 5 mm from the ostium (Figure 3). 

**Figure 4:**
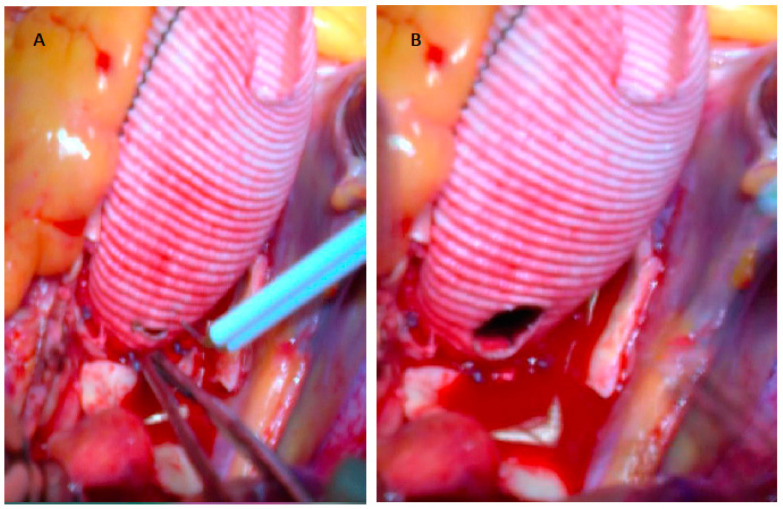
Openings performed in the vascular conduit proportionate to the diameter of the left coronary ostium. Initial incision with electrocauterium (**A**). Final opening (**B**).

After the insertion of the composite graft into the aortic annulus, we passe outside the aortic annulus two or three additional valvular stitches in correspondence of the non-coronary sinus and of the left-coronary sinus, to reinforce it and to avoid possible pseudoaneurysm formation. In addition, we passe over the composite graft the three commissural stitches that we use at the beginning to suspend the aortic valve. At this point of the surgical procedure, using an electrocautery, we prepare in the vascular conduit the opening to attach the left coronary ostium in an end-to-side fashion with running 5-0 polypropylene suture (“button technique”). To avoid distortion of the left main artery, we paid attention: (a) to align the conduit to the distal ascending aorta; (b) to leave the left coronary ostium in place and to lay the conduit on the left coronary ostium; (c) to sign the correct position of the left coronary ostium in the vascular conduit; (d) to make the openings performed in the vascular conduit proportionate to the diameter of the coronary ostium (Figure 4).

**Figure 5:**
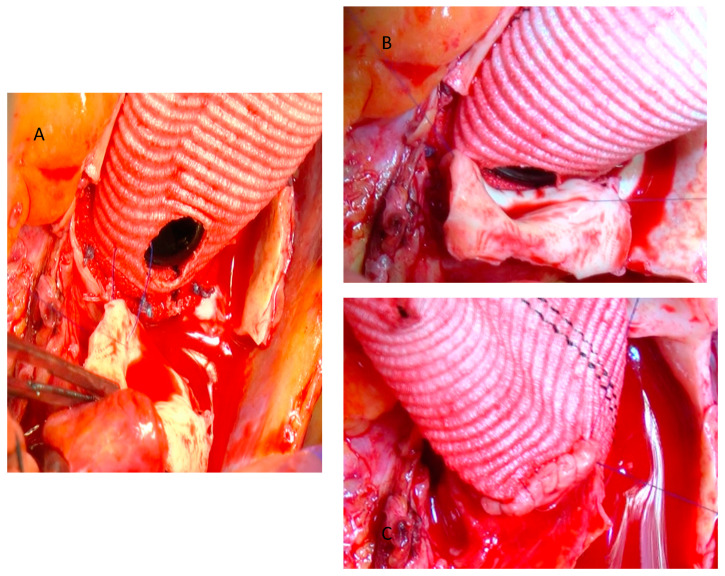
Anastomosis between left coronary ostium and vascular conduit (**A**–**C**).

In mechanical Bentall, we start to make the opening about 4–5 mm from the ring of the valve prosthesis (about two folds of the vascular conduit); in bio-Bentall, we keep higher than mechanical prosthesis, in order to avoid damaging of the base of the cusp. In addition, in order to achieve an optimal and very accurate anastomosis, we paid attention: (a) to use a 5/0 polypropylene stitch, at least 75 cm long, with a small needle; (b) to start suturing on the side of the second surgeon to perform the lower part of the anastomosis first; (c) to pass the stitches almost at the level of the ostium of the left main artery not to leave segments of the aortic wall (possible subsequent dilatation due to aortic pathology); (d) not to bring the ostium close to the vascular conduit if the passage of the stitches for the entire lower part is not completed; and (e) to develop a correct tension of the polypropylene thread (Figure 5). 

When coronary ostia wall is degenerated, the suture of the left coronary ostium to the vascular conduit can be reinforced with a small strip of Teflon (about 3 mm large). Before suturing the right coronary ostium, we performed the distal anastomosis of the vascular graft to the transected aorta using a continuous 4-0 polypropylene suture routinely reinforced with a Teflon strip along the entire perianastomotic suture to control surgical bleeding. Particular care is taken to measure the length of the vascular graft. In this regard, it is important to keep in mind that the length of the medial portion of the ascending aorta is smaller than the lateral one. Furthermore, the prosthesis should not be stretched excessively to avoid excessive tension on the distal anastomosis and allow for a minimum of elasticity during ventricular systole. In addition, it will be helpful to keep a few millimeters of vascular graft available in case there are surgical problems in the anastomosis. During the distal anastomosis, we pay attention: (a) to start suturing on the side of the second surgeon to perform the lower part of the anastomosis first; (b) to pass the first stitches keeping the conduit 3–4 cm away from the aorta to facilitate the passage of the other stitches; (c) to bring the vascular conduit closer to the aorta after having performed the posterior positioning of the stitches. At this point the second surgeon, with the surgical forceps, takes the vascular conduit at the level of the first stitch while the first surgeon performs the same maneuver on his side. Then the 4-0 polypropylene suture is gently pulled from both sides, using a thin hook, avoiding tearing the aortic wall. Three-to-five millimeters of vascular prosthesis should be placed inside the aortic wall.

**Figure 6:**
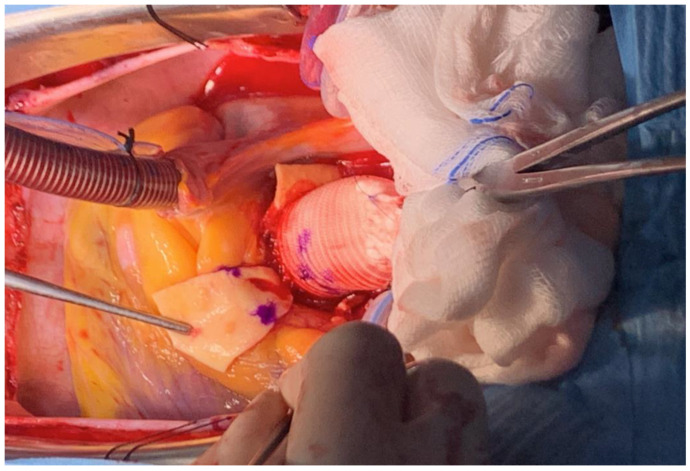
The choice of the correct position of the right coronary ostium on the vascular conduit.

At this point of the surgical procedure, using an electrocautery, we prepare in the vascular conduit the opening to attach the right coronary ostium in an end-to side fashion with running 5-0 polypropylene suture (“*button technique*”). To avoid distortions of the right coronary artery, in this phase of the operation it is very important to follow these rules: (a) to ask the perfusionist to leave blood volume in the right ventricle to better visualize the correct position of the right coronary ostium on the conduit; (b) to sign the correct position of the coronary ostium on the vascular conduit and mark two lateral points both on the prosthesis and on the walls of the ostium to prevent accidental twisting of the ostium itself (Figure 6); (c) to perform the opening in the vascular conduit proportionate to the diameter of the coronary ostium. In order to achieve an optimal and very accurate anastomosis it is important: (a) to use a 5/0 polypropylene stitch, at least 75 cm long, with a small needle; (b) to start suturing on the side of the second operator to perform the lower part of the anastomosis first; (c) to pass the stitches almost at the level of the ostium of the right coronary artery not to leave segments of the aortic wall (possible subsequent dilatation due to aortic pathology); (d) not to bring the ostium close to the vascular conduit if the passage of the stitches for the entire lower part is not completed; (e) to develop the right entity of the tension of the polypropylene thread. When the wall of the right coronary ostium is degenerated, the suture to the vascular graft is reinforced with a small strip of Teflon (about 3 mm large). Finally, using a Ticron 2-0 stitch reinforced with two pledget of Teflon, the aortic vent is put in the vascular conduit for the de-airing. Particular attention must be paid during de-airing to avoid excessive manipulation of the heart causing traction of the coronary ostia.

The remaining part of the operation does not require any particular technical measures compared to other types of cardiac surgery.

### 2.5. Statistical Analysis

Statistical analysis was performed using Stat View 4.5 program (SAS Institute Inc., Abacus Concepts, Berkeley, CA, USA). Continuous values were expressed as mean value plus or minus one standard deviation. To analyze potential risk factors at the univariate analysis for the main postoperative cardiac-related complications, i.e., hospital death and acute heart failure with or without renal impairment, we applied chi-squared or Fisher’s exact tests for the measurements of the categorial variables, and the unpaired Student’s *t*-test for the measurements of the continuous variables. The following preoperative and perioperative variables were analyzed: age, gender, NYHA class, rhythm, co-morbidities (chronic pulmonary disease, chronic renal dysfunction, and peripheral arteriopathy), cardio-vascular risk factors (smoking habit, dyslipidemia, diabetes mellitus, obesity, and arterial hypertension), type of aortic valve pathology, the presence of the ischemic coronary artery disease with or without previously documented myocardial infarction, perioperative echocardiographic parameters mentioned above, the mean duration of the extracorporeal circulation and the aortic cross-clamp, need for associated procedure, i.e., CABG. The variables that reached *p* values less than 0.1 were included in a multivariable analysis in order to detect independent predictors of postoperative major cardiac-related complications and death. All *p*-values < 0.05 were considered statistically significant.

## 3. Results

The mean age of patients with a mechanical valved conduits was 57 ± 14 years, the mean age of patients with a biological valved conduit was 76 ± 10 years (*p* < 0.001). As expected, extracorporeal circulation (180 ± 60 min vs. 115 ± 28 min, *p* < 0.0001) and aortic cross-clamp (150 ± 27 min. vs. 99 ± 23 min., *p* < 0.0001) times were longer in the associated procedures in comparison with isolated Bentall operations. Euroscore-2 was higher in patients who underwent associated procedures than isolated one (6.5% ± 1.5% vs. 4.6% ± 0.3%, *p* = 0.04). Operative mortality was 1.2% (*n* = 5), specifically it was 0.69% (*n* = 2/289) for isolated Bentall operation, 2.65% (*n* = 3/113) for combined procedures (*p* = 0.001). One 45-year-old men patient undergone mechanical prosthesis valved conduit implantation died early for septic shock related to a gluteal abscess (0.44%, *n* = 1/226). An 85-year-old patient (man) died for postoperative respiratory failure associated with a septic shock; another 74-year-old male patient died for hyperacute pulmonary failure requiring ECMO, another 82-year-old patient (female) died for postoperative acute heart failure and multiple organ failure; the other one 69-year-old patient (female) died for heart failure at the weaning from cardiopulmonary bypass. Thirty-eight patients (9.5%) developed low cardiac output syndrome. At the univariate analysis, the risk factors for operative death and postoperative heart failure were the advanced age at the time of operation (77 vs. 64 years; *p* = 0.0007), the preoperative pulmonary arterial hypertension (44 ± 13 vs. 32 ± 7 mmHg; *p* = 0.006), longer extracorporeal circulation (169 ± 61 min vs. 124 ± 42 min) and aortic cross-clamp (133 ± 46 min vs. 107 ± 33 min) times (*p* < 0.001, for both measurements), and concomitant associated CABG (*p* < 0.0001). At the Logistic regression, the independent predictors remained the preoperative higher value of pulmonary arterial hypertension (HR 2.0), and the longer cardiopulmonary bypass (HR 2.0) and aortic cross-clamp (HR 1.9) times (*p* < 0.05, for all measurements). There was no cases of early valve thrombosis or coronary ostia obstruction after the operation. One patient had early postoperative bleeding from the implantation of the right coronary ostium; two patients experienced this complication from the implantation of the left coronary ostium. There was one case of intraoperative right coronary ostia dissection treated with the ligation of the coronary ostium and coronary artery bypass on the right coronary artery. 

## 4. Discussion

**Figure 7:**
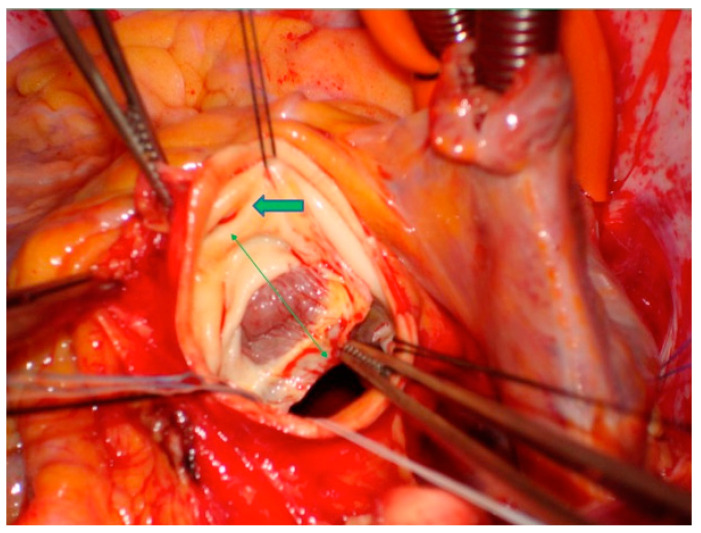
Evidence of cardiac muscle in transparency without endothelium at the level of the right coronary sinus. Green arrows: right coronary ostium.

Bentall operation remains the most used operation and it is not limited to particular valve morphology or particular conditions such as syndromic patients, bicuspid aortic valve, coronary ostia dislocation, and altered morphology of the aortic wall [18,19]. Experienced centers performed the Bentall operation with very good postoperative results. Additional coronary artery disease could influence late outcomes [12,20]. Specific expedients in surgical technique can greatly improve the results. In our center, in-hospital mortality was 0.69% for isolated Bentall operations, 2.65% for combined operations, and 0.44% in younger patients. These values of mortality and morbidity are lower than those reported in other studies [2,21]. It is important not to underestimate the fact that the cross-clamp time and cardiopulmonary bypass time in our study population were lower than that reported in other studies [2]. This is related not only to the number of procedures performed per year (about 23 operations per year), and therefore to the center’s surgical experience, but also to our personal specific surgical expedients developed during a twenty-year period. For these reasons, we gave surgical indication to patients affected by aortic dilation of 50 mm also in absence of other risk factors, in order to avoid rupture and dissection. The earliest surgical indication to treat aneurysms, with a diameter of 50 mm, underlies two important aspects that we want to underline: first, that the risk of rupture/dissection/sudden death for aortic dilations between 45 and 50 mm is reported to be between 4 and 7% per year, and therefore appears higher (2–3 times) compared to the mortality observed in our study; second, that the operative risk for the treatment of acute aortic complications is 15–20 times higher when the surgical intervention is performed in emergency. This decision was also supported by important studies [11,12,16] performed in our center that questioned the role of the aortic diameter in order to prevent catastrophic complications like rupture and acute dissection. Our surgical indication was also based on etiological parameters and on morphological features, i.e., coronary ostia dislocation and prolapse, quality of the aortic wall, asymmetric dilation of Valsalva sinus/sinuses, aortic-annulus disjunction, and presence of the heart muscle through the aortic annulus without endothelium (Figure 7) at the level of right- or non-coronary sinus. We found these last two features in approximately 10% of the operated patients.

Our good clinical results may have depended on particular technical expedient that we detected during the operation. Reading the scientific literature, the most-deadly complications after Bentall operations are related to the uncorrected position of the coronary ostia anastomoses, which can lead stenosis, torsion, dissection, laceration, or onset of late pseudoaneurysm/s. In particular, coronary ostium distortion is a rare but important complication after the reimplantation. Its incidence has been evaluated between 5% and 6% in patients undergoing Bentall–De Bono operation [22]. In a systematic review of the Bentall operation it is reported an early mortality of 5.6%, with 5.9% of this related with an early coronary ostial obstruction [23]. Several mechanisms of coronary ostia obstruction have been described, including a direct damage during their manipulation or during the delivery of cardioplegia, and issues related with sutures of the coronary button [24]. Careful reimplantation of the coronary button is very important in order to avoid this complication [25]. Particular techniques to facilitate tension-free button attachment has been reported [26]. According with this personal approach to Bentall–De Bono operation, we suggest to performed at the beginning of the preparation, only a minimal mobilization of both coronary ostia. In addition, it is important that the site of right coronary re-implantation should be determined only after anastomosis of the Dacron graft to the distal native ascending aorta. Even then, the precise site is determined by temporarily filling the heart. Finally, the right orientation of the suture of the ostia must be signed with a colored signal for the correct initiation of the anastomosis on the opening of the vascular conduit. Following these precise steps, there were no cases of coronary ostia obstruction or kinking after the operation in our surgical experience. In addition, we reduce the risk of coronary pseudoaneurysm formation excising the coronary ostia together with a ‘collar’-like remnant of the aortic wall (extending 5 mm from the ostium). Particular attention was taken during the anastomosis, gently favoring the juxta between the coronary ostia and prosthetic vascular tissue, and pulling the polypropylene thread of the anastomosis for the control of the hemostasis with a hook after the first three-to-four steps with 5-0 polypropylene suture. This expedient reduces the risk of peri-anastomotic coronary ostia bleeding. We experienced early postoperative bleeding from the right coronary ostium anastomosis only in one case; from the left coronary ostium anastomosis in two cases. The same approach was used to realize the distal anastomosis between the prosthetic vascular graft and the aortic tissue with the 4-0 polypropylene, with the interposition of a Teflon strip outside the entire perianastomotic suture. This expedient reduces the risk of pseudoaneurysm formation at the level of the distal anastomosis and at the level of the coronary ostia. Another important observation in the analysis of our study population was that we did not find cases of early thrombosis in patients who implanted a biological valved conduit. In our opinion this is the advantage in the use of a straight and rigid vascular conduit (Intervascular, Datascope Corp., Wayne, NJ, USA). Using this type of vascular conduit, we minimize the space between the aortic walls and valve leaflets during ventricular systole, likely resulting in minimal blood flow turbulence and, consequently, in a low risk of thrombus formation. In addition, this type of vascular conduit in our opinion reduces the risk of coronary ostia distortion or kinking because it allows a better and precise coronary ostia anastomosis localization.

The main limitations of the study in question are represented by the fact that it is a personal surgical experience, and the study is individual retrospective.

## 5. Conclusions

Although the Bentall–De Bono operation has been widely used for years, specific expedients in surgical technique can greatly improve the results. In my twenty-year personal experience, the Bentall operation, with the adoption of a series of technical measures, can be performed with low rate of operative mortality and complications. For this reason, we have modified our surgical indication for all types of ascending aortic aneurysms, not based solely and mainly on the size of the aneurysm, and now we think that it is time to change surgical guidelines.

## Data Availability

They are attached as supplement data.

## Supplementary Materials

The following supporting information can be downloaded at: https://www.mdpi.com/article/10.3390/jcm12237330/s1. Video S1: VIDEO pubb-sut ostio cor sx.

## References

[1] Bentall H., De Bono A. (1968). A technique for complete replacement of the ascending aorta. Thorax.

[2] Mookhoek A., Korteland N.M., Arabkhani B., Lansac I., Bekkers J.A., Bogers A.J., Takkenberg J.J. (2016). Bentall Procedure: A Systematic Review and Meta-Analysis. Ann. Thorac. Surg..

[3] Castrovinci S., Tian D.H., Murana G., Cefarelli M., Berretta P., Alfonsi J., Yan T.D., Di Bartolomeo R., Di Eusanio M. (2015). Aortic Root Replacement With Biological Valved Conduits. Ann. Thorac. Surg..

[4] Urbanski P.P., Heinz N., Zhan X., Hijazi H., Zacher M., Diegeler A. (2010). Modified bio-Bentall procedure: 10-year experience. Eur. J. Cardiothorac. Surg..

[5] Di Marco L., Pacini D., Pantaleo A., Leone A., Barberio G., Marinelli G., Di Bartolomeo R. (2016). Composite valve graft implantation for the treatment of aortic valve and root disease: Results in 1045 patients. J. Thorac. Cardiovasc. Surg..

[6] Lechiancole A., Celiento M., Isola M., Gatti G., Melina G., Vendramin I., Battistella C., Pappalardo A., Sinatra R., Bortolotti U. (2019). Modified Bentall procedure: Mechanical vs biological valved conduits in patients older than 65 years. Int. J. Cardiol..

[7] Stefanelli G., Pirro F., Macchione A., Bellisario A., Weltert L. (2020). Long-term follow-up after Bentall operation using a stentless Shelhigh NR-2000 bio-conduit. J. Card. Surg..

[8] Katselis C., Samanidis G., Papasotiriou A., Kriaras I., Antoniou T., Khoury M., Michalis A., Perreas K. (2017). Long-Term Results after Modified Bentall Operation in 200 Patients. J. Heart Valve Dis..

[9] Celiento M., Ravenni G., Margaryan R., Ferrari G., Blasi S., Pratali S., Bortolotti U. (2016). The Modified Bentall Procedure: A Single-Institution Experience in 249 Patients with a Maximum Follow Up of 21.5 Years. J. Heart Valve Dis..

[10] Dhurandhar V., Parikh R., Saxena A., Vallely M.P., Wilson M.K., Black D.A., Tran L., Reid C.M., Bannon P.G. (2016). The Aortic Root Replacement Procedure: 12-year Experience from the Australian and New Zealand Society of Cardiac and Thoracic Surgeons Database. Heart Lung. Circ..

[11] Nardi P., Ruvolo G. (2016). Current surgical indications to surgical repair of the aneurysms of the ascending aorta. J. Vasc. Endovasc. Surg..

[12] Nardi P., Pisano C., Bassano C., Bertoldo F., Salvati A.C., Buioni D., Trombetti D., Asta L., Scognamiglio M., Altieri C. (2022). Bentall Operation: Early Surgical Results, Seven-Year Outcomes, and Risk Factors Analysis. Int. J. Env. Res. Public. Health.

[13] Pisano C., D’Amico F., Balistreri C.R., Vacirca S.R., Nardi P., Altieri C., Scioli M.G., Bertoldo F., Santo L., Bellisario D. (2020). Biomechanical properties and histomorphometric features of aortic tissue in patients with or without bicuspid aortic valve. J. Thorac. Dis..

[14] Scola L., Di Maggio F.M., Vaccarino L., Bova M., Forte G.I., Pisano C., Candore G., Colonna-Romano G., Lio D., Ruvolo G. (2014). Role of TGF-β pathway polymorphisms in sporadic thoracic aortic aneurysm: rs900 TGF-β2 is a marker of differential gender susceptibility. Mediat. Inflamm..

[15] Pisano C., Balistreri C.R., Nardi P., Altieri C., Bertoldo F., Buioni D., Ruvolo G. (2020). Risk of aortic dissection in patients with ascending aorta aneurysm: A new biological, morphological and biochemical networkbehind the aortic diameter. Vessel. Plus..

[16] Bassano C., Vacirca S.R., Colella D., Bertoldo F., Pugliese M., Ferrante M.S., Ruvolo G. (2018). Is the diameter of the aorta a safe parameter for cardiac surgery indication in aortic aneurysm? Proceedings of XXIX SICCH Meeting, 23–25 November 2018, Rome. J. Cardiovasc. Med..

[17] Vahanian A., Beyersdorf F., Praz F., Milojevic M., Baldus S., Bauersachs J., Capodanno D., Conradi L., De Bonis M., De Paulis R. (2022). 2021 ESC/EACTS Guidelines for the management of valvular heart disease. Eur. Heart J..

[18] Arabkhani B., Klautz R.J.M., de Heer F., De Kerchove L., El Khoury G., Lansac E., Schäfers H.J., El-Hamamsy I., Lenoir M., Aramendi J.I. (2023). A multicentre, propensity score matched analysis comparing a valve-sparing approach to valve replacement in aortic root aneurysm: Insight from the AVIATOR database. Eur. J. Cardiothorac. Surg..

[19] Wallen T., Habertheuer A., Bavaria J.E., Hughes G.C., Badhwar V., Jacobs J.P., Yerokun B., Thibault D., Milewski K., Desai N. (2019). Elective Aortic Root Replacement in North America: Analysis of STS Adult Cardiac Surgery Database. Ann. Thorac. Surg..

[20] Stamou S.C., Williams M.L., Gunn T.M., Hagberg R.C., Lobdell K.W., Kouchoukos N.T. (2015). Aortic root surgery in the United States: A report from the Society of Thoracic Surgeons database. J. Thorac. Cardiovasc. Surg..

[21] Akins C.W., Miller D.C., Turina M.I., Kouchoukos N.T., Blackstone E.H., Grunkemeier G.L., Takkenberg J.J., David T.E., Butchart E.G., Adams D.H. (2008). Guidelines for reporting mortality and morbidity after cardiac valve interventions. J. Thorac. Cardiovasc. Surg..

[22] Ziakas A.G., Economou F.I., Charokopos N.A., Pitsis A.A., Parharidou D.G., Papadopoulos T.I., Parharidis G.E. (2010). Coronary ostial stenosis after aortic valve replacement. Tex. Heart Inst. J..

[23] Adamson C., Rocchiccioli P., Brogan R., Berry C., Ford T.J. (2019). Post-operative myocardial infarction following aortic root surgery with coronary reimplantation: A case series treated with percutaneous coronary intervention. Eur. Heart J. Case Rep..

[24] Westaby S., Katsumata T., Vaccari G. (2000). Aortic root replacement with coronary button re-implantation: Low risk and predictable outcome. Eur. J. Cardiothorac. Surg..

[25] Sultan I., Komlo C.M., Bavaria J.E. (2016). How I teach a valve-sparing root replacement. Ann. Thorac. Surg..

[26] Urbanski P.P. (2000). Aortic root replacement with coronary button reimplantation. Eur. J. Cardiothorac. Surg..

